# The Clinical Mentorship Program: a mixed-methods evaluation of a community-based medical education pathway program for high school students

**DOI:** 10.1186/s12909-026-09852-x

**Published:** 2026-07-04

**Authors:** Victor M. Hunt, Jamie Cheng, Weston C. de Lomba, Sarita Warrier

**Affiliations:** 1 The Warren Alpert Medical School, Brown University, Providence, Rhode Island USA; 2 Annenberg Institute, Brown University, Providence, Rhode Island USA

**Keywords:** Medical education, Clinical education, Pathway program, Mentorship, Shadowing, Underrepresented students, Health equity

## Abstract

Students from low-income and underrepresented backgrounds face structural barriers to entering medicine, including limited access to early clinical exposure, physician guidance, and professional networks. To address these disparities, medical students at the Warren Alpert Medical School of Brown University created a year-long Clinical Mentorship Program (CMP) for high school students in Woonsocket, Rhode Island. The intervention combined monthly after-school instruction on basic science content and clinical skills by medical students with structured hospital-based shadowing and guided discussion with resident physicians. Using a mixed-methods pre-post design, the intervention evaluated changes in educational motivation, clinical self-efficacy, medical knowledge, awareness of other academic enrichment programs, and physician access and comfort. High school students demonstrated significant gains in motivation to attend college, understanding of cardiopulmonary anatomy and physiology, understanding of physician roles, comfort interacting with physicians, self-reported physician access, and awareness of local educational opportunities. Qualitative findings highlighted the importance of experiential learning, guided shadowing, a clearer understanding of medical careers, and persistent structural barriers. These findings suggest that community-based programs that leverage local resources, networks, and infrastructure to combine medical education with guided clinical exposure may be feasible entry points into the medical pipeline for students confronting complex socioeconomic disadvantages.

## Introduction

Medical school admission is predicated not only on academic ability, but also by financial resources, access to information and opportunity, and personal investment from a supportive network of family and mentors. This is evident by a disproportionate number of U.S. medical students coming from affluent households, with nearly 75% of medical students representing the top two household-income quintiles. Meanwhile, students from low-income backgrounds remain markedly underrepresented in medicine. At the extremes of the income distribution, 24% came from the top 5% of household earners, whereas only 6.1% came from the lowest income quintile [1]. More recent work has likewise shown declining representation of first-generation and low-income students within the national medical student body [2].

Similarly, early exposure to medicine as a potential career is common among students who later matriculate into medical school. Nearly two-thirds of medical students report deciding to pursue medicine before or during high school. Among medical student respondents to the Matriculating Student Questionnaire, approximately 10% reported prior participation in middle school premedical programming and 10% in high school premedical programming, while 13% reported summer research experience during high school [3]. These early experiences may facilitate access to physician networks, mentorship, and clinical opportunities while increasing awareness of the profession, reinforcing career-related self-efficacy, and solidifying medicine as a potential career pathway. In addition to boosting confidence and academic motivation, [4] mentorship in the health professions provides access to the implicit knowledge and cultural competencies that define the art of medicine [5]. Through interactions with mentors and physician role models, students gain insight into educational pathways, professional expectations, and the unwritten norms that shape entry into the medical profession.

These patterns likely reflect broader socioeconomic differences in the educational and professional opportunities available across communities. Variability in school funding, enrichment programming, healthcare-related academic opportunities, and access to professional networks may contribute to unequal exposure to medicine as a career pathway, particularly among students enrolled in lower-resourced public schools. For example, Woonsocket, a historically distressed city in northern Rhode Island, is a community with persistent socioeconomic challenges. With a per-capita income of $30,593, 19.7% of residents live in relative poverty, and only 18.6% of residents aged 25 years or older have attained a bachelor’s degree or higher [6]. Although much of the literature has focused on pre-medical and medical students, the benefits of mentorship and exposure to authentic clinical environments are well documented across the educational continuum [7–10]. These observations highlight the importance of creating accessible opportunities for students in underserved communities early in their educational trajectories. Woonsocket High School (WHS) was therefore selected as the site of an intervention designed to address these gaps.

In addition to serving a student population facing educational and economic barriers, WHS is located near Landmark Medical Center (LMC), a community hospital with an internal medicine residency program affiliated with New York Medical College. To address barriers related to cost, access, and limited exposure to healthcare careers, medical students at the Warren Alpert Medical School of Brown University developed the Clinical Mentorship Program (CMP), a year-long after-school program for WHS students that combined interactive lectures, clinical skills instruction, and hospital-based shadowing. The aim of the CMP was to evaluate whether participation was associated with changes in educational motivation, medical knowledge, awareness of educational pathways, comfort in clinical settings, and access to physician role models, and to describe how students experienced the program qualitatively.

## Methodology

### Study design

This study was approved by the Brown University Institutional Review Board (STUDY0000063: Clinical Mentorship Program) before data collection began and was conducted in accordance with the Declaration of Helsinki and applicable institutional and federal guidelines for research involving human participants. Written informed consent was obtained from parents or legal guardians and written informed assent was obtained from student participants.

The CMP was designed as a longitudinal mixed-methods intervention combining didactic instruction, clinical skills training, and guided hospital-based shadowing. The evaluation focused on four domains: (1) educational and career motivation, (2) clinical self-efficacy and medical knowledge, (3) awareness of local educational pathway programs, and (4) comfort interacting with and access to physicians. Because the intervention centered on scheduled shadowing and interactive didactics, the term mentorship in this manuscript refers to program-facilitated access to resident and physician role models rather than to an intensive, independently sustained mentoring relationship outside the program, although some residents and students later reported professional working relationships beyond the conclusion of the program.

Student recruitment occurred through flyers posted throughout the school, teacher announcements, and email distribution through school listservs. Participation was open to all students, with no academic or eligibility requirements for enrollment. Because the program was extracurricular and offered no academic credit, attendance at lectures and shadowing sessions was voluntary.

Lectures were hosted monthly throughout the academic year, each lasting approximately two hours. The introductory lecture provided an overview of topics commonly addressed through formal mentorship relationships, including educational timelines, the role of undergraduate experiences in medical school matriculation, physician debt and future earning potential across medical specialties, communication and empathy in patient care, and the core tenets of medical ethics. It also introduced students to locally available academic, healthcare, and research opportunities relevant to students interested in pursuing careers in medicine, such as Brown’s Program in Liberal Medical Education (PLME), HealthCORE, and Brown Summer High School (BSHS).

Subsequent lectures focused on foundational medical science and clinical skills training. Emphasizing the cardiovascular and pulmonary systems, the curriculum introduced anatomy, physiology, and common pathology of the heart and lungs. Through interactive exercises, students practiced professional introductions, medical interviewing, components of the history of present illness (HPI), review of systems (ROS), focused cardiopulmonary physical examination skills, and the oral case presentation.

Parallel to the classroom curriculum, students participated in shadowing sessions at LMC. Each participant was paired with a resident physician from the New York Medical College internal medicine residency program for the academic year. Resident physicians were provided learning objectives and discussion prompts aligned with the after-school didactics so that each shadowing session remained goal-oriented and corresponded with content from the most recent monthly lecture. These interactions were designed to provide guided clinical exposure, role modeling, and opportunities for questions about clinical work and training. The program did not require mentor-mentee meetings outside scheduled shadowing activities. A summary of the CMP curriculum is provided in Table 1. 

**Table 1:** Clinical Mentorship Program curriculum

Session	Title	Interactive Lecture - Learning Objectives	Shadowing Session - Learning Objectives
1	Introduction to Clinical Mentorship Program	Outline the program’s objectives and expectations for participation. Introduce core principles of bioethics and their relevance in clinical care. Describe the structure of a patient encounter and how clinical reasoning and medical knowledge contribute to forming a differential diagnosis. Practice introducing oneself professionally in a clinical setting. Review the structure of the HPI and conduct a basic HPI interview.	Review program expectations, hospital professionalism, and key principles of HIPAA. *Introduce and discuss Sect. 3 (HPI) of the Medical Interview Checklist. *Encourage students to practice professional introductions during patient encounters. For example: “*Hello*,* my name is Christina. I’m a junior at Woonsocket High School in the Clinical Mentorship Program*,* and I’m here shadowing Dr. Ramirez. Is it alright if I join her during your visit?”*
2	Medical Interview: Medical, Family, and Social Histories	Review and practice patient introductions and the HPI. Discuss the structure and purpose of the medical, family, and social histories. Explore the role of social determinants of health in shaping patient outcomes. Integrate all components to conduct a full medical interview, including the introduction, HPI, and medical, family, and social histories.	*Encourage students to continue practicing their professional introduction during each new patient encounter. *Prompt students to actively identify components of the HPI during clinical conversations, using the HPI Cheatsheet as a reference. *To build confidence, clinician can consider prompting students—after initial encounters—to return and practice confirming the patient’s medical, family, and social history; this exercise is for learning purposes only and should not be used for clinical documentation.
3	Cardiovascular System: Anatomy, Physiology, and Disease	Describe the basic anatomy and physiology of the cardiovascular system, including the structure and function of the heart and major vessels. Explain the pathway of blood circulation through the heart, lungs, and systemic circulation. Identify key signs and symptoms associated with common cardiovascular conditions (e.g., chest pain, shortness of breath, palpitations). Apply foundational knowledge to interpret simple cardiovascular cases and discuss possible diagnoses.	Discuss with clinician how they apply their knowledge of cardiovascular anatomy and physiology when establishing a relevant differential diagnosis. Identify how common cardiovascular symptoms (e.g., chest pain, shortness of breath, palpitations) present in real clinical encounters and discuss how these guide diagnostic reasoning.
4	Cardiovascular System: Review of Systems and Physical Exam	Introduce the cardiovascular ROS and physical exam. Identify and recite common cardiovascular ROS symptoms. Associate common cardiovascular conditions, to relevant physical exam findings. Perform a basic cardiovascular physical exam, including assessment of heart sounds and pulses, using mannequins, audio sets, and fellow consenting students.	Before the session, review Sect. 7 (ROS: general, cardiovascular) of the Medical Interview Checklist and the Cardiopulmonary Exam Checklist. *Review the cardiovascular ROS through interactive quizzing. During patient encounters, student should identify pertinent cardiovascular ROS findings. *Discuss common cardiovascular conditions and examine their associated physical exam findings.
5	Pulmonary System: Anatomy, Physiology, and Disease	Describe the basic anatomy and physiology of the pulmonary system, including the structure and function of the lungs and major airways. Explain the mechanics of respiration and gas exchange within the alveoli. Identify key signs and symptoms associated with common pulmonary conditions (e.g., cough, shortness of breath, wheezing). Apply foundational knowledge to interpret simple pulmonary cases and discuss possible diagnoses.	Discuss with clinician how they apply their knowledge of pulmonary anatomy and physiology when establishing a relevant differential diagnosis. Identify how common pulmonary symptoms (e.g., cough, shortness of breath, wheezing) present in real clinical encounters and discuss how these guide diagnostic reasoning. *Interpret a chest X-ray from a patient with a cardiopulmonary diagnosis, highlighting key clinical correlations
6	Pulmonary System: Review of Systems and Physical Exam	Introduce the pulmonary ROS and physical exam. Identify and recite common pulmonary ROS symptoms. Associate common pulmonary conditions, such as pneumonia, COPD, and asthma, with relevant physical exam findings. Perform a basic pulmonary physical exam, including auscultation of breath sounds, using mannequins, audio sets, and fellow consenting students.	Before the session, review Sect. 7 (ROS: pulmonary) of the Medical Interview Checklist and the Cardiopulmonary Exam Checklist. *Review the pulmonary ROS through interactive quizzing. During patient encounters, student should identify pertinent pulmonary ROS findings. *Discuss common pulmonary conditions and examine their associated physical exam findings. *Interpret a chest X-ray from a patient with a cardiopulmonary diagnosis, highlighting key clinical correlations
7	Objective Structured Clinical Examination (OSCE): Review Session	Briefly review previously learned content and clinical skills, including the medical interviews, ROS, physical exam. Integrate knowledge from prior sessions to approach simulated patient cases in a systematic and confident manner. Introduce how to summarize and present this content as an oral presentation. Practice delivering clear and concise oral case presentations that synthesize key clinical findings. Identify personal areas for improvement and receive feedback to guide final preparation for the OSCE.	Practice conducting an oral presentation for a patient case.
8	OSCE: Final Assessment	In the final capstone session, students will participate in an OSCE where they will demonstrate the skills they developed throughout the program. Each student will conduct a simulated patient encounter (a medical student or the student’s clinical mentor) that includes taking a complete medical history, performing a focused physical examination, and delivering an oral presentation summarizing their findings and clinical reasoning.	Before the session, the student should reflect on their experiences during the clinical shadowing component of the CMP, considering key takeaways, moments of growth, and areas for continued learning. The student is encouraged to engage in a debriefing conversation with their physician mentor, sharing what they found most meaningful and discussing any insights gained. They are encouraged to express appreciation for the mentor’s guidance and support, and to inquire about future opportunities for longitudinal collaboration, if they are inclined to do so.

Abbreviated outline of the eight-session CMP curriculum delivered to high school students by medical student instructors and clinical mentors, highlighting the learning objectives of didactic instruction and shadowing sessions, respectively

*Abbreviations*: *HPI* history of present illness, *ROS* review of systems, *HIPAA* Health Insurance Portability and Accountability Act, *OSCE* objective structured clinical examination, *COPD* chronic obstructive pulmonary disease

* Starred items represent suggestions or instructions for mentors during shadowing sessions

Of note, while formal mentorship typically involves sustained developmental guidance, the shadowing and other forms of early clinical exposure offered through the CMP were intended to provide access to role modeling, professional insight, and situated learning. For students from underserved backgrounds, access to transient and longitudinal mentorship may both be limited. Accordingly, this study emphasizes a community-based intervention centered on medical education, guided clinical exposure, and structured access to physician and resident role models, rather than intensive longitudinal career mentorship extended beyond the schedule program activities throughout the academic year. However, in addition to facilitating clinical exposure and professional connections, the CMP incorporated topics commonly addressed through formal mentorship relationships, including those listed above.

### Participants and procedures

Participants were WHS students who enrolled in the CMP during the 2024–2025 academic year. Participation in the program, surveys, and group reflection session was voluntary. Surveys were administered through Qualtrics at the first and final program sessions. A total of 27 students completed the pre-program survey and 6 completed the post-program survey. Eligibility for free or reduced-price lunch, as designated by the school through the federal school meal program, was used as a proxy for lower socioeconomic status. Demographic characteristics of survey respondents are summarized in Table 2.

**Table 2:** Demographic characteristics of CMP survey respondents (*n* = 27)

Demographic Characteristic	*n* (%)
Grade Level	
12th grade	13 (48.1)
11th grade	4 (14.8)
10th grade	5 (18.5)
9th grade	5 (18.5)
Sex	
Female	21 (80.8)
Male	5 (19.2)
No Response	1 (3.7)
Race	
Black or African American	8 (33.3)
White	6 (25.0)
Multiracial	5 (20.8)
Asian/Indian/Pacific Islander	3 (12.5)
Indigenous or Alaska Native	1 (4.2)
Middle Eastern or North African	1 (4.2)
No Response	3 (11.1)
Ethnicity	
Non-Hispanic/Latino/Spanish	15 (55.6)
Hispanic/Latino/Spanish	12 (44.4)
Socioeconomic Status	
Qualifies for free/reduced lunch	17 (63.0)
Does not qualify for free/reduced lunch	10 (37.0)
Parental Education Level	
High school/GED or less	16 (59.3)
Some college or above	11 (40.7)
Family Physician Status	
Has physician in family (immediate or extended)	2 (7.4)
No physician in family	25 (92.6)

Demographics of survey respondents (*n* = 27), primarily 12th graders, with a majority identifying as female and representing diverse racial and ethnic backgrounds. Most respondents qualified for free or reduced lunch, had parents with a high school education or less, and reported no physician in their family

Free/reduced-price lunch eligibility was used as a proxy for lower socioeconomic status

### Data collection

#### CMP survey

Data collection instruments were designed to align with the program’s educational objectives. The survey was administered via Qualtrics and included five-point Likert-scale items and multiple-choice questions. Items were organized into four domains: motivation, clinical self-efficacy and medical knowledge, awareness of educational programs, and physician access and mentorship. The survey item labeled “number of physician mentors” is interpreted in this manuscript as a self-reported indicator of physician access or available role models; because the term was not formally defined within the survey, the item should not be interpreted as proof of a formal longitudinal mentoring relationship, but rather a subjective measure on the breadth of a students’ physician network.

The program, including the survey instrument, was piloted with a small group of students during the previous academic year. Feedback from the pilot informed revisions to improve item clarity and alignment with curricular content. The survey questionnaire and interview guide are provided in Supplementary Items.

#### CMP interview guide

In addition to the survey, a semi-structured group reflection guide was developed for the end-of-program discussion. Questions asked students to describe memorable program experiences, whether the program changed how they thought about pursuing medicine, how physician guidance or shadowing influenced their understanding of medicine, what barriers students like them face when pursuing medicine, and what they would change about the program.

To strengthen analytic transparency, Table 3 maps the core interview prompts to example codes and the final themes reported in the Results section.

**Table 3:** Linkage between interview prompts, example codes, and final qualitative themes

Interview prompt	Example codes	Resulting theme
Was there a moment during the program that stood out to you?	Hands-on learning; shadowing; patient interaction; observing clinical practice	Experiential learning and guided shadowing enhanced engagement and understanding
Before CMP, what were your thoughts about pursuing medicine? Have they changed?	Testing the waters; increased openness; stronger interest; greater clarity	The program increased clarity about medical careers
Based on your experience in the CMP, how important is physician guidance or mentorship?	Role models; physician guidance; networking; learning what doctors actually do	Physician access and role modeling were valuable
What challenges do students like you face when pursuing medicine?	Financial barriers; lack of exposure; uncertainty about pathway; lack of connections	Persistent structural barriers and limited opportunities remained salient
If you could change one thing about CMP, what would it be?	More opportunities; expansion; sustained access; more shadowing	Persistent structural barriers and limited opportunities remained salient

Linkage between semi-structured reflection prompts, representative participant codes, and final themes identified through thematic analysis. Themes were developed using Braun and Clarke’s six-phase framework and illustrate how students perceived the educational, experiential, mentorship, and structural dimensions of the CMP

### Data analysis

#### Quantitative analysis

Survey responses were exported from Qualtrics for analysis. All statistical analyses were conducted using Python (version 3.9), with Pandas for data cleaning, NumPy for numerical operations, SciPy for statistical testing, and Matplotlib and Seaborn for data visualization. Given the non-randomized study design, the small post-survey sample size (*n* = 6), and the ordinal nature of the Likert-scale data, non-parametric tests were used for group comparisons. Mann-Whitney U tests were used for ordinal items, and Fisher’s exact test was used for categorical awareness questions as appropriate. Effect sizes were calculated to aid interpretation of practical significance.

#### Qualitative analysis

The transcript from the group reflection session was analyzed using Braun and Clarke’s six-phase thematic analysis framework [11]. To strengthen trustworthiness, two reviewers independently read the transcript, generated initial codes, clustered codes into candidate themes, and resolved discrepancies through consensus discussion. Themes were then refined, named, and reported with illustrative quotations.

## Results

### Survey results

Participants reported mixed changes in motivation across academic and medical career pathways. There was no statistically significant increase in academic motivation overall (*p* = 0.182), motivation to work in healthcare (*p* = 0.520), motivation to apply to medical school (*p* = 0.350), or motivation to become a physician (*p* = 0.801). Motivation to attend college increased significantly from pre- to post-program (*p* = 0.020), with a large effect size.

Perceived confidence in specific clinical skills, including medical interviewing and physical examination, did not increase significantly, although point estimates generally trended in a positive direction. By contrast, students demonstrated significant gains in perceived cardiopulmonary anatomy and physiology knowledge (*p* = 0.016) and in understanding physician roles and responsibilities (*p* = 0.046).

Awareness of all three educational pathway programs increased significantly: PLME, HealthCORE, and BSHS. However, interest in applying to these programs did not change significantly, suggesting that increased awareness did not necessarily translate into immediate aspirational alignment or application intent.

**Fig. 1:**
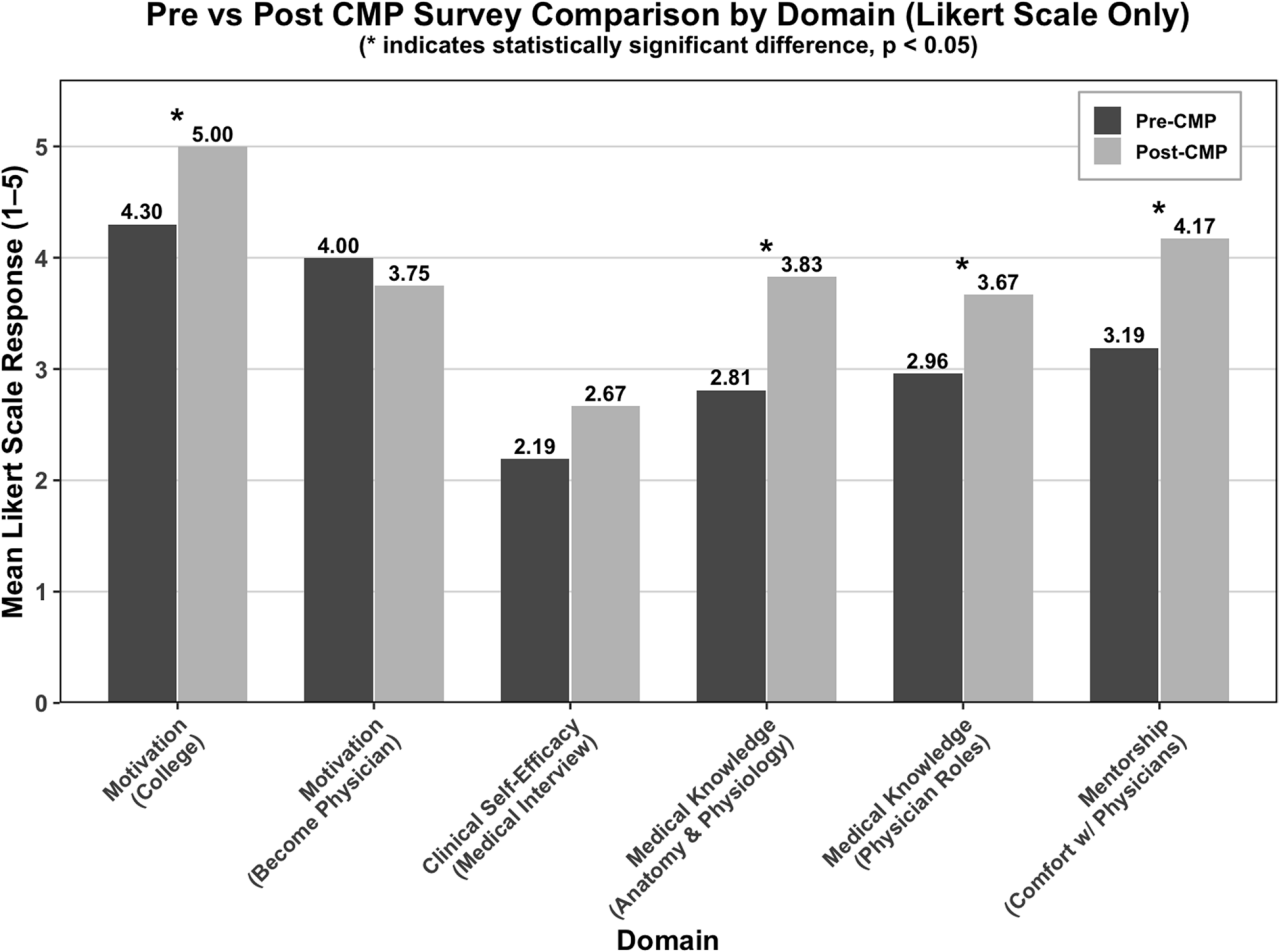
Likert-scale average student responses for pre- and post-CMP survey questionnaires across the main domains. Significant differences were detected in selected motivation, medical knowledge, and physician-access outcomes

Students reported a significant increase in comfort interacting with physicians (*p* = 0.007) and in their self-reported number of physician mentors (mean increase from 0.67 to 1.83, *p* = 0.003). Other community-related items, including comfort interacting with patients, sense of belonging in healthcare settings, and perceived importance of mentorship, did not change significantly. Findings are summarized in Table 4 and Fig. 1.

**Table 4:** Quantitative outcomes of CMP survey across developmental domains

Domain	Variable	*p*-value	Effect Size (Cohen’s d)
Motivation	Academic motivation†	0.182	0.479
Motivation	College motivation†	0.020*	1.091**
Motivation	Motivation to work in healthcare†	0.520	0.018
Motivation	Motivation to apply to medical school†	0.350	0.210
Motivation	Motivation to become a physician†	0.801	-0.463
Clinical Self-Efficacy	Medical interview confidence†	0.136	0.504
Clinical Self-Efficacy	Review of systems confidence†	0.302	0.172
Clinical Self-Efficacy	Physical exam confidence†	0.471	0.037
Medical Knowledge	Anatomy & physiology knowledge†	0.016*	1.144**
Medical Knowledge	Understanding of physician roles†	0.046*	0.847**
Program Awareness	PLME awareness†	< 0.001*	—
Program Awareness	HealthCORE awareness‡	0.005*	—
Program Awareness	BSHS awareness‡	0.027*	—
Program Awareness	Interest in applying to PLME‡	0.135	—
Program Awareness	Interest in applying to HealthCore‡	0.529	—
Program Awareness	Interest in applying to BSHS‡	0.613	—
Physician Access	Comfort interacting with physicians†	0.007*	1.328**
Physician Access	Comfort interacting with patients†	0.911	-0.564
Physician Access	Sense of belonging in a healthcare setting†	0.340	0.178
Physician Access	Perceived value of mentorship†	0.257	0.474
Physician Access	Self-reported number of physician mentors/contacts† (mean increase 0.67 to 1.83)	0.003*	1.518**

Effect sizes of measured domains from the survey. Cohen’s* d* > 0.8 indicates a large effect. Anatomy & physiology knowledge and comfort working with physicians had the largest effect sizes among the domains measured

* Statistically significant. ** Large effect size. † Mann-Whitney U test. ‡ Fisher’s exact test

### Reflection session results

Thematic analysis of the reflection session yielded four closely related themes that aligned with the interview guide: (1) experiential learning and guided shadowing enhanced engagement and understanding, (2) the program increased clarity about medical careers, (3) physician access and role modeling were valuable, and (4) students perceived persistent structural barriers and limited opportunities.

#### Theme 1: experiential learning and guided shadowing enhanced engagement and understanding

Participants consistently described the program’s hands-on components, particularly mock interviews and clinical shadowing, as the most memorable and educational aspects of the CMP. These experiences made medicine feel more concrete and helped students connect classroom learning to clinical practice.

*“The medical interviews, those are really fun. Those are hands on and I really enjoyed that.”*

*“Actually asking questions and trying to diagnose, that was really cool.”*

*“I like the part when we actually went into shadow and saw how it is in practice…”*

#### Theme 2: the program increased clarity about medical careers

Students often described the CMP as helping them better understand what a career in medicine looks like and whether it fit their interests. Their comments suggested increased clarity and openness toward medicine, even when survey measures of medical career motivation remained statistically unchanged.

*“This program… encouraged me and motivated me to definitely pursue medicine more.”*

*“I initially joined… just to test the waters. But now I feel like I'm more open to the medical field.”*

*“I wanted to pursue medicine, but not as much as I do now.”*

#### Theme 3: physician access and role modeling were valuable

Students emphasized the value of interacting with physicians and resident physicians who could show them what the profession actually looks like. Their comments were more consistent with guided exposure, role modeling, and question-and-answer support than with intensive longitudinal mentoring outside the program.

*“If I hadn't shadowed, I would be a lot more in the dark… He was really cool, showing me what it is to be a physician.”*

*“If you don’t have a mentorship, you're going to be pretty unprepared for it.”*

#### Theme 4: persistent structural barriers and limited opportunities remained salient

Participants described structural barriers that extended beyond the CMP itself, including lack of exposure, limited networking opportunities, uncertainty about the path to medicine, and financial concerns. These comments contextualized the program’s perceived value and underscored why students viewed expanded opportunities as important.

*“Public schools have a lot less opportunities than private schools. We don't have access to learning more about medical stuff.”*

*“The major one would be networking, like having those connections.”*

*“It costs a lot to go to a medical school.”*

*“I think… I'm not completely sure what the process entails.”*

## Discussion

Taken together, the quantitative and qualitative findings suggest that the CMP functioned primarily as an early exposure and medical education program rather than that which measurably changed all aspects of participating students’ professional identity within a single year. The strongest quantitative signals were increased college motivation, increased perceived medical knowledge, greater comfort interacting with physicians, increased awareness of pathway programs, and higher self-reported physician access.

These findings are consistent with the program’s structure, which emphasized instruction, guided shadowing, and exposure to clinical environments. Prior work has shown that even short-term clinical immersion programs can broaden students’ perspectives on healthcare and strengthen confidence in their career aspirations [12], suggesting that meaningful educational benefits may be achievable through relatively low-resource interventions. The present findings extend this literature by demonstrating similar gains among high school students from an underserved community through a program that combined didactic instruction with year-long clinical exposure.

The qualitative findings help explain why some outcomes changed more than others. Students most often highlighted experiential elements such as mock interviews and shadowing and described these activities as making the field of medicine more interesting and tangible. This supports the interpretation that a principal contribution of the CMP was to reduce informational distance between students and the profession by providing concrete experiences, language, and role models.

Importantly, students clearly valued access to physicians and resident physicians, and the program appears to have increased students’ perceived ability to identify physicians they could approach for future mentorship and guidance. However, the present study did not measure the depth, duration, or continuity of mentoring relationships outside scheduled program activities. Accordingly, the CMP should be interpreted as providing guided shadowing, physician access, and role modeling, with mentorship as an emerging or partial feature rather than a fully characterized longitudinal outcome.

Participants’ comments about networking, limited school-based opportunities, cost, and uncertainty about the educational pathway also help situate the findings within a broader equity context. For these students, the CMP did not simply deliver content; it also addressed an access problem by creating experiences that many participants described as otherwise unavailable in their educational setting. This likely helps explain why awareness of pathway programs and comfort interacting with physicians improved even when motivation to become a physician did not increase significantly.

The stability of most medicine-specific motivation items should not necessarily be interpreted as a programmatic failure. Because enrollment was voluntary, many students likely entered the CMP with pre-existing interest in medicine. In that context, the program may have served more to clarify, reinforce, or test existing interests than to create them de novo. Qualitative comments support this interpretation, with students often describing greater clarity or openness rather than a simple increase in enthusiasm. Future iterations of the CMP could strengthen its mentorship component by training resident physicians around developmental mentorship goals, incorporating structured career conversations, and building formal opportunities for longitudinal follow-up beyond shadowing sessions. More systematic attendance tracking for missed sessions would also help guide stakeholders in understanding barriers to consistent participation.

Overall, the CMP appears at least feasible as a community-based model for combining early medical education, guided clinical exposure, and access to physician role models for high school students from underserved backgrounds. The present findings support feasibility and short-term educational value, but do not establish long-term effects on college enrollment, persistence in pre-health pathways, or eventual entry into medicine.

### Limitations

This study has several limitations. First, surveys were anonymous and voluntary, so responses could not be matched at the individual level. The post-survey sample was also small (*n* = 6), limiting statistical power and precision. Several outcomes demonstrated large effect sizes, suggesting potentially meaningful impacts that warrant further evaluation in larger cohorts. Although the present study was not designed to assess long-term outcomes, prior mentorship and pathway programs have reported sustained benefits, including continued mentor-mentee engagement and persistence on pre-health tracks [13]. The lower post-program response rate was partly attributable to a school-wide end-of-year event that coincided with the final session, when the post-survey was administered.

Second, participation in this after-school program was voluntary, and attendance across sessions was not systematically analyzed. As a result, the study cannot determine how dose of exposure influenced outcomes, nor can it definitively explain why some students did not continue attending. Competing obligations such as work, family responsibilities, transportation, and competing extracurricular activities may all have contributed. The absence of a control group also reflects broader challenges in healthcare education outreach research, where most published evaluations lack matched comparison groups, limiting causal inference and attribution of observed outcomes directly to program participation [14].

Third, most outcomes were based on self-report. These measures are useful for feasibility work, but they provide limited evidence regarding actual skill acquisition or long-term behavioral change. The survey item regarding number of physician mentors was also not formally defined for respondents and may have captured a range of interpretations.

Fourth, the qualitative findings were derived from a single end-of-program group reflection session and should be interpreted as descriptive rather than exhaustive. Although two reviewers independently coded the transcript and resolved discrepancies through consensus, the themes reflect a small cohort at one institution. Future studies would benefit from stronger follow-up, objective knowledge assessments, attendance tracking, matched longitudinal data, and, where feasible, comparison groups. 

## Data Availability

The data supporting this study’s findings are available from the corresponding author upon reasonable request. Restrictions apply due to privacy and ethical considerations. No publicly accessible repository is available for these data.
